# VCTE Overestimates Liver Fibrosis due to Abdominal–Truncal Adiposity and Not Hepatic Steatosis: A Case Report

**DOI:** 10.1155/2024/7938701

**Published:** 2024-10-28

**Authors:** Jordan S. Woodard, Jena Velji-Ibrahim, Jay Alden, Gary A. Abrams

**Affiliations:** ^1^University of Kentucky Healthcare, Department of Medicine, Lexington, Kentucky, USA; ^2^Prisma Health-Upstate, Department of Medicine, Greenville, South Carolina, USA; ^3^Prisma Health-Upstate, Department of Pathology, Greenville, South Carolina, USA

## Abstract

Vibration-controlled transient elastography (VCTE) is used for the noninvasive assessment of liver fibrosis. We present a case of significant weight loss over 1 year, resulting in a marked improvement in liver stiffness suggesting a decrease in liver fibrosis from stage 4 (cirrhosis) to stage 2 (moderate fibrosis) notably without a change in the grade of hepatic steatosis. The improvement in two stages of fibrosis over this short time frame is due to the overestimation of liver stiffness in a subject with class 3 obesity and not due to the resolution of fibrosis. Therefore, this case highlights that BMI, due to excess subcutaneous abdominal adipose tissue and not intrahepatic lipid accumulation, can cause a significant overestimation of liver fibrosis with VCTE.

## 1. Introduction

Metabolic dysfunction-associated steatotic liver disease (MASLD) is the leading cause of chronic liver disease worldwide, and its prevalence continues to rise rapidly [1]. The presence of liver fibrosis is a crucial predictor of liver-related and overall mortality. Therefore, accurate assessment of liver fibrosis is essential for MASLD patients. The AGA screening guidelines for MASLD fibrosis are evolving to include earlier use of noninvasive testing in patients who are at high risk for fibrosis including those with either prediabetes, type 2 diabetes, or at least two metabolic risk factors [2].

Vibration-controlled transient elastography (VCTE) using FibroScan® is a noninvasive tool to assess liver fat accumulation with the controlled attenuation parameter (CAP) score as well as stage of fibrosis based on liver stiffness, measured in kilopascals (kPa) [3]. Previous studies have shown that VCTE has high diagnostic accuracy in the assessment of steatosis and liver fibrosis; particularly, it has high clinical validity in diagnosing advanced fibrosis [4]. VCTE can accurately diagnose advanced fibrosis (F3) and cirrhosis (F4) with an area under the receiver operating characteristic (AUROC) of 0.687 for F3-4 and an AUROC of 0.984 for F4 [4]. However, confounding factors such as high BMI and severe hepatic steatosis can diminish its accuracy and lead to overestimation of fibrosis [5, 6]. Here, we present the case of a 48-year-old female who had significant weight loss resulting in improvement in VCTE from stage 4 to stage 2 fibrosis with no change in the grade of hepatic steatosis.

## 2. Case Presentation

**Figure 1:**
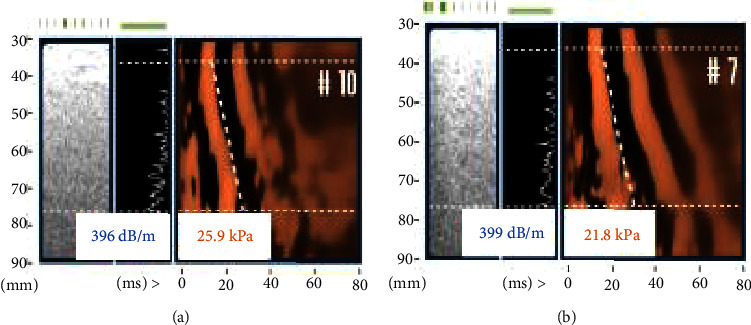
FibroScan® at initial evaluation. At initial evaluation, FibroScan® was obtained in two separate rib spaces ((a) and (b)). CAP and kPa from the two sites were averaged. Results revealed an average CAP score of 398 dB/m, suggestive of grade 3 steatosis, and an average liver stiffness of 23.9 kPa, suggestive of cirrhosis (F4). CAP–controlled attenuation parameter; dB/m–decibel per meter; kPa–kilopascal.

A 48-year-old female with a history of class 3 obesity, MASLD, asthma, pulmonary fibrosis, and hyperlipidemia was referred to gastroenterology for irritable bowel syndrome. She had no history of illicit drug use and was prescribed ondansetron 8 mg three times a day as needed, hyoscyamine 0.125 mg four times a day as needed, montelukast 10 mg daily, dexlansoprazole 60 mg daily, and albuterol 90 mcg/budesonide 80 mcg daily. Her BMI was 40.3 kg/m^2^ (250 pounds), and her waist circumference was 41.5 inches. She had steatosis documented on imaging 4 years prior. AST and ALT were unremarkable at 21 IU/L and 22 IU/L, respectively. The initial FIB-4 score was 1.43, and she was agreeable to VCTE for MASLD screening in the setting of an indeterminate-risk FIB-4 score. She was contacted in advance of VCTE to fast for at least three hours, as recommended by Echosens, the manufacturer of the FibroScan® 530, and she confirmed that she did indeed fast at the time of her scan. VCTE was performed in the office using the XL probe with measurements in two separate rib spaces (Figure 1). Her average CAP score was 398 dB/m and liver stiffness was 23.9 kPa suggesting steatosis grade S3 and stage 4 fibrosis (cirrhosis). She exhibited no signs or symptoms of cirrhosis or portal hypertension. While liver biopsy was recommended, she declined in favor of lifestyle modification and repeat VCTE in 1 year.

**Figure 2:**
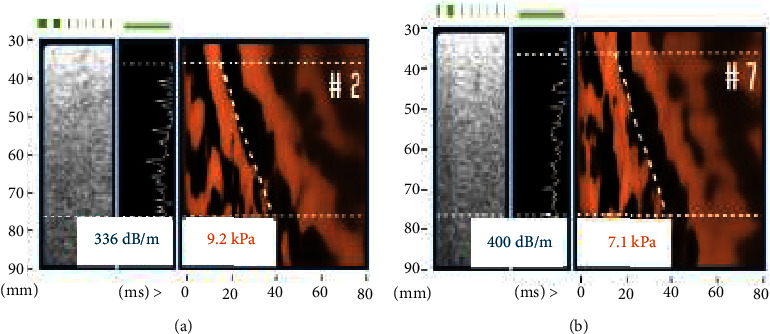
FibroScan® at one-year follow-up. FibroScan® was repeated in two separate rib spaces ((a) and (b)) after 1 year of weight loss. CAP and kPa from the two sites were averaged. An average CAP score of 368 dB/m continues to suggest grade 3 steatosis. Average liver stiffness is 8.2 kPa suggesting significant fibrosis (F2), a major change from the initial FibroScan® (Figure 1). CAP–controlled attenuation parameter; dB/m–decibel per meter; kPa–kilopascal.

The patient was started on once-weekly subcutaneous semaglutide 0.25 mg and titrated to a dose of 2.4 mg after 17 weeks by her primary care doctor while also making lifestyle modifications. At one year follow-up, she had a 59-pound weight loss (24% body weight). Her BMI decreased from 40.3 kg/m^2^ to 30.8 kg/m^2^, and her waist circumference decreased from 41.5 to 33 inches. AST and ALT values remained within normal limits at 16 IU/L and 13 IU/L, respectively. A repeat FIB-4 score decreased to 1.21, placing her in the low-risk category for advanced fibrosis (stages 3 or 4 fibrosis). VCTE was repeated in two rib spaces using the XL probe (Figure 2). The average CAP score was 368 dB/m, and liver stiffness was 8.2 kPa, now supporting stage 2 fibrosis (significant fibrosis). She continued lifestyle modification with a plan for repeat VCTE screening in 2 years.

## 3. Discussion

Here, we describe a case of significant weight loss with VCTE suggesting resolution of cirrhosis (stage 4 fibrosis) to stage 2 fibrosis within 1 year. Both scans were completed by the same experienced technician while the patient was fasting for at least 3 hours. The rapid degree of improvement in our patient's fibrosis is most likely due to overestimation of the initial liver stiffness measurement (LSM) due to class 3 obesity as regression of 2 stages of fibrosis in 1 year is unlikely. Lassailly and colleagues [7] followed three patients with cirrhosis after bariatric surgery and showed none of the patients had regression at 1 year and only one patient had regression of 1 fibrosis stage in 5 years. This supports the low likelihood of fibrosis regression in our patient with suspected cirrhosis on initial VCTE.

Our patient did not have evidence of cirrhosis or portal hypertension on examination. However, patients who do have evidence of cirrhosis are at risk of hepatic decompensation when they undergo rapid weight loss. For example, a case report described a patient with MASH cirrhosis who was treated with semaglutide and who developed ascites and hepatic encephalopathy due to rapid weight loss [8]. In that case, cessation of semaglutide, aggressive nutrition, and restoration of lost weight resulted in liver recompensation.

Increased skin-to-capsule distance (SCD) is a known risk factor for fibrosis overestimation by VCTE [9]. The XL probe has been used to improve accuracy in patients with obesity. In our case, the XL probe was utilized, and although not ideal, it is the probe suggested to be used in subjects with a BMI greater than 32 kg/m2 when using the FibroScan 530 model. The XL probe may not be fully effective in compensating for higher BMI. Failure of the XL probe is supported by Fang and colleagues [10] who demonstrated that 38% of patients with a BMI > 28 kg/m^2^ had a major fibrosis discrepancy, and Myers and colleagues [11] showed 4-fold discordance with BMI ≥ 40 kg/m^2^. Recently, Echosens has developed a “SmartExam” that increases the skin-to-capsule distance, and this improves the estimation of liver fibrosis in obese individuals [12].

Elevated CAP scores have also been associated with the overestimation of fibrosis by VCTE [13, 14]. Our case demonstrates that despite a 24% weight loss, with our patient having class 3 obesity to class 1 obesity, and a 20% decrease in abdominal circumference, her CAP score continued to suggest grade 3 steatosis (> 66%). To date, the effects of semaglutide in subjects with MASLD and fibrosis have shown a 42% reduction in liver fat, inflammation, and hepatocellular injury but no significant improvement in liver fibrosis after 72 weeks of therapy [15]. Therefore, it is doubtful semaglutide had any direct effect on improving liver fibrosis. Thus, the overestimation of liver stiffness suggesting cirrhosis was primarily influenced by BMI and excess truncal adiposity and not the degree of hepatic fat accumulation.

While the liver biopsy is the gold standard to stage liver fibrosis, it is impractical to perform biopsies on everyone with MASLD due to the costs and potential complications. Therefore, screening for liver fibrosis in MASLD has moved away from obtaining a liver biopsy, and the recent AGA guidelines recommend screening with noninvasive testing that include VCTE, the enhanced liver fibrosis (ELF) test, or magnetic resonance elastography (MRE) [2].

## 4. Conclusion

It is important to recognize that the XL probe can mitigate VCTE failures in obesity, but overestimation of liver fibrosis may lead to an unnecessary liver biopsy. Additional testing with the ELF test or MRE before proceeding to a liver biopsy may be warranted especially in class 3 obesity. Further studies are needed to confirm that liver stiffness discordance with fibrosis on biopsy is primarily from excess subcutaneous adipose and not due to the severity of intrahepatic fat.

## Data Availability

No data were used to support this study.
